# Temperature-dependent excitonic superuid plasma frequency evolution in an excitonic insulator, Ta_2_NiSe_5_

**DOI:** 10.1038/s41598-018-30430-9

**Published:** 2018-08-10

**Authors:** Yu-Seong Seo, Man Jin Eom, Jun Sung Kim, Chang-Jong Kang, Byung Il Min, Jungseek Hwang

**Affiliations:** 1 Department of Physics, Sungkyunkwan University, Suwon, Gyeonggi-do 16419 Republic of Korea; 2 Department of Physics, Pohang University of Science and Technology, Pohang, 37673 Republic of Korea

## Abstract

An interesting van der Waals material, Ta_2_NiSe_5_ has been known one of strong excitonic insulator candidates since it has very small or zero bandgap and can have a strong exciton binding energy because of its quasi-one-dimensional crystal structure. Here we investigate a single crystal Ta_2_NiSe_5_ using optical spectroscopy. Ta_2_NiSe_5_ has quasi-one-dimensional chains along the *a*-axis. We have obtained anisotropic optical properties of a single crystal Ta_2_NiSe_5_ along the *a*- and *c*-axes. The measured *a*- and *c*-axis optical conductivities exhibit large anisotropic electronic and phononic properties. With regard to the *a*-axis optical conductivity, a sharp peak near 3050 cm^−1^ at 9 K, with a well-defined optical gap (${{\boldsymbol{\Delta }}}_{{\boldsymbol{o}}{\boldsymbol{p}}}^{{\boldsymbol{E}}{\boldsymbol{I}}}\,{\boldsymbol{\simeq }}$ 1800 cm^−1^) and a strong temperature-dependence, is observed. With an increase in temperature, this peak broadens and the optical energy gap closes around ∼325 K (${{\boldsymbol{T}}}_{{\boldsymbol{c}}}^{{\boldsymbol{E}}{\boldsymbol{I}}}$). The spectral weight redistribution with respect to the frequency and temperature indicates that the normalized optical energy gap ($(\,{{\boldsymbol{\Delta }}}_{{\boldsymbol{o}}{\boldsymbol{p}}}^{{\boldsymbol{E}}{\boldsymbol{I}}}({\boldsymbol{T}})/\,{{\boldsymbol{\Delta }}}_{{\boldsymbol{o}}{\boldsymbol{p}}}^{{\boldsymbol{E}}{\boldsymbol{I}}}{\bf{(0)}})$) is ${\bf{1}}{\boldsymbol{-}}{({\boldsymbol{T}}/{{\boldsymbol{T}}}_{{\boldsymbol{c}}}^{{\boldsymbol{E}}{\boldsymbol{I}}})}^{{\bf{2}}}$. The temperature-dependent superfluid plasma frequency of the excitonic condensation in Ta_2_NiSe_5_ has been determined from measured optical data. Our study may pave new avenues in the future research on excitonic insulators.

## Introduction

Excitonic insulators (EI), proposed in the 1960s^1–3^, are novel materials exhibiting correlated electronic phases and have attracted the interest of several experimental and theoretical condensed matter physics groups. An EI has a condensation phase of excitons (or electron-hole pairs) as its ground state for a specific condition (${E}_{b} > {E}_{g}$)^3^, where *E*_*b*_ and *E*_*g*_ are the binding energy and bandgap, respectively. In the EI phase, superfluidity of neutral electron-hole pairs occurs^4^. EI systems are either semiconductors with small bandgaps or semi-metals with small overlaps between the conduction and valence bands^3^. The excitonic condensation in semiconductors occurs through a Bose-Einstein condensation process, while the condensation in semi-metals occurs through the Bardeen-Cooper-Schrieffer (BCS) process^5,6^. Typically, chalcogenide compounds are known to form one group of EIs. Several studies have been performed on Ta_2_NiSe_5_, which is one of transition metal chalcogenides^7–16^. Ta_2_NiSe_5_ has quasi-one-dimensional chains along the *a*-axis^7,8^. An angle-resolve photoemission (ARPES) study on Ta_2_NiSe_5_ showed that the top of the valence band at the Γ-point flattened at temperatures below its structural transition temperature (${T}_{c}^{Str}$ = 325 K), and this flat band was interpreted as an excitonic insulating ground state of condensed electron-hole pairs of Ta 5 *d*-electrons and Ni 3 *d*- and Se 4 *p*-holes^9^. A recent study also shows that Ta_2_NiSe_5_ is a zero-gap semiconductor, with a transitions to an EI occurring near 326 K (referred as the onset temperature (${T}_{c}^{EI}$))^15^. There was another very recent ellipsometry spectroscopic study on Ta_2_NiSe_5_^16^; the authors claimed that exciton-phonon complexes in Ta_2_NiS_5_ and Ta_2_NiSe_5_ are confirmed and their observation agrees with the hypothesis of an excitonic insulator ground state. In this article, we provide a new set of anisotropic optical data of Ta_2_NiSe_5_ obtained using a different optical spectroscopy technique from the ellipsometry technique. We observed the temperature-dependent evolution of the excitonic insulator energy gap (or excitonic condensation gap) of Ta_2_NiSe_5_. Furthermore, we extracted a very important physical quantity, the excitonic superfluid plasma frequency, of Ta_2_NiSe_5_ from the measured optical data.

## Anisotropic reflectance spectra

**Figure 1:**
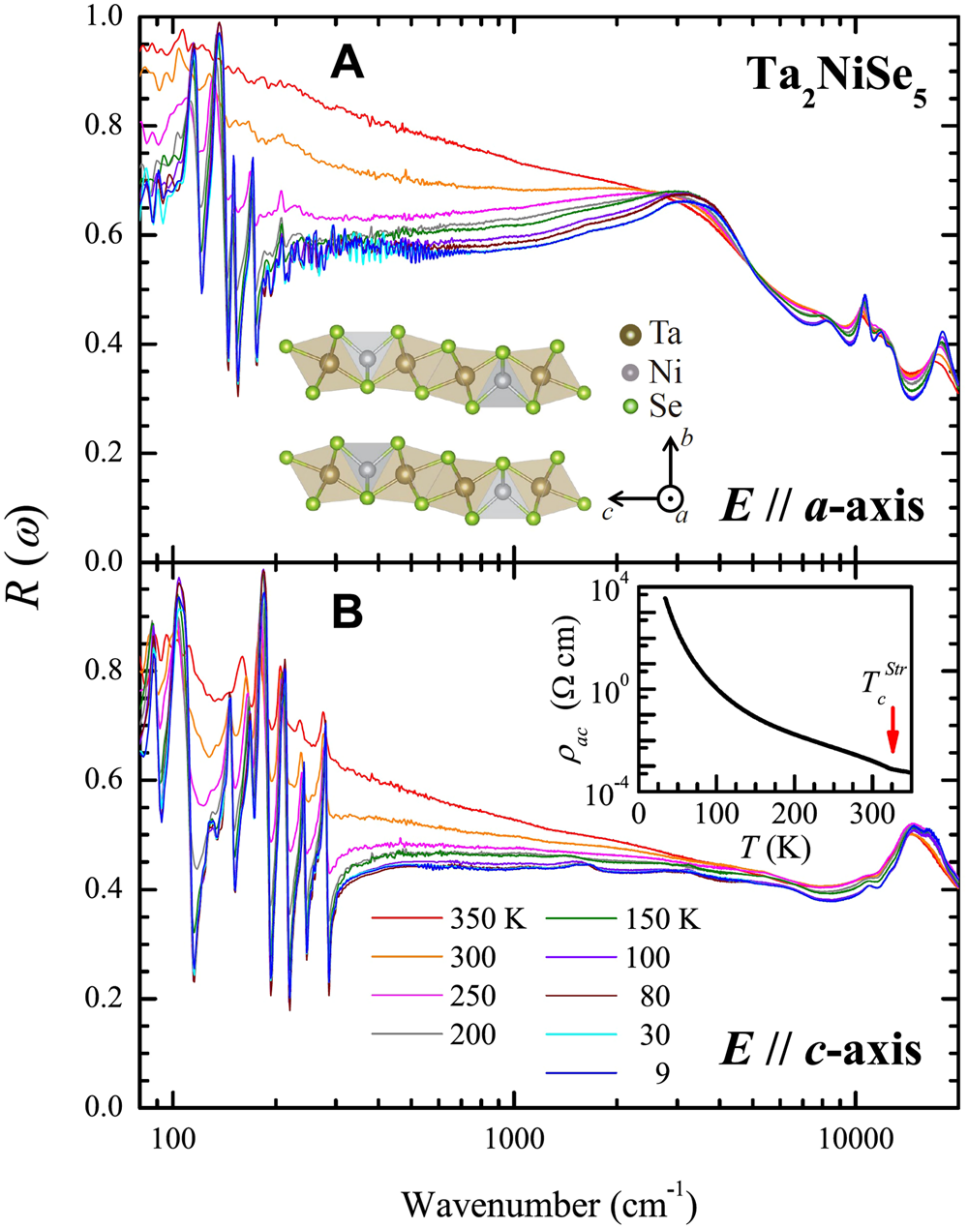
Anisotropic reflectance spectra of Ta_2_NiSe_5_ along *a*- and *c*- axes. The measured reflectance spectra of Ta_2_NiSe_5_ along the *a*- and *c*-axes are displayed in (**A**) and (**B**) respectively. The spectra were recorded at selected temperatures ranging from 9 to 350 K. The crystal structure of Ta_2_NiSe_5_ is shown in the inset of (**A**), while the measured dc resistivity of *ac*-plane of Ta_2_NiSe_5_ is depicted in the inset of (**B**).

We present the temperature-dependent anisotropic optical properties of single crystal Ta_2_NiSe_5_, recorded along two different crystal orientations (*a*- and *c*-axis) using a conventional optical spectroscopic technique. (refer to the Method) Fig. 1(A and B) show the measured reflectance spectra of a single crystal Ta_2_NiSe_5_ along the *a*- and *c*-axis respectively. There was a significant difference in the electronic and phononic properties along the two different crystal axis orientations (*a*- and *c*-axes) as we expected. For Ta_2_NiSe_5_, the quasi-one-dimensional chains are along the *a*-axis. Several sharp peaks were observed in the *a*-axis reflectance spectra at low temperatures, with similar strong temperature-dependent behaviors. The reflectance below ∼2700 cm^−1^ increases gradually with temperature and above the frequency a peak centered near 3200 cm^−1^ grows with lowering the temperature. This behavior is a typical signature of optical gap formation. The phonon modes seem to be screened at high temperatures above 300 K. The inset in Fig. 1(A) depicts the crystal structure of Ta_2_NiSe_5_, which has a layered structure with the *b*-axis as the stack axis. In contrast to the *a*-axis reflectance spectra, the *c*-axis reflectance spectra displays a rather monotonic temperature-dependence, with the reflectance being gradually suppressed over a wide spectral range from 80 to ∼15,000 cm^−1^ with the lowering of the temperature. A set of peaks with weak intensity and narrow spectral widths appear at low temperatures. The physical origin of these new set of peaks are not clear yet. The experimentally measured dc resistivity of the *ac*-plane of Ta_2_NiSe_5_ is displayed in the inset of Fig. 1(B). An anomaly in the dc resistivity associated with the structural phase transition temperature (${T}_{c}^{Str}$) was observed close to 322 K^8^. Below the ${T}_{c}^{Str}$, we also observed the splitting of a phonon mode centered around 160 cm^−1^, which indicates that the structural phase transition clearly takes place.

## Anisotropic optical conductivity

**Figure 2:**
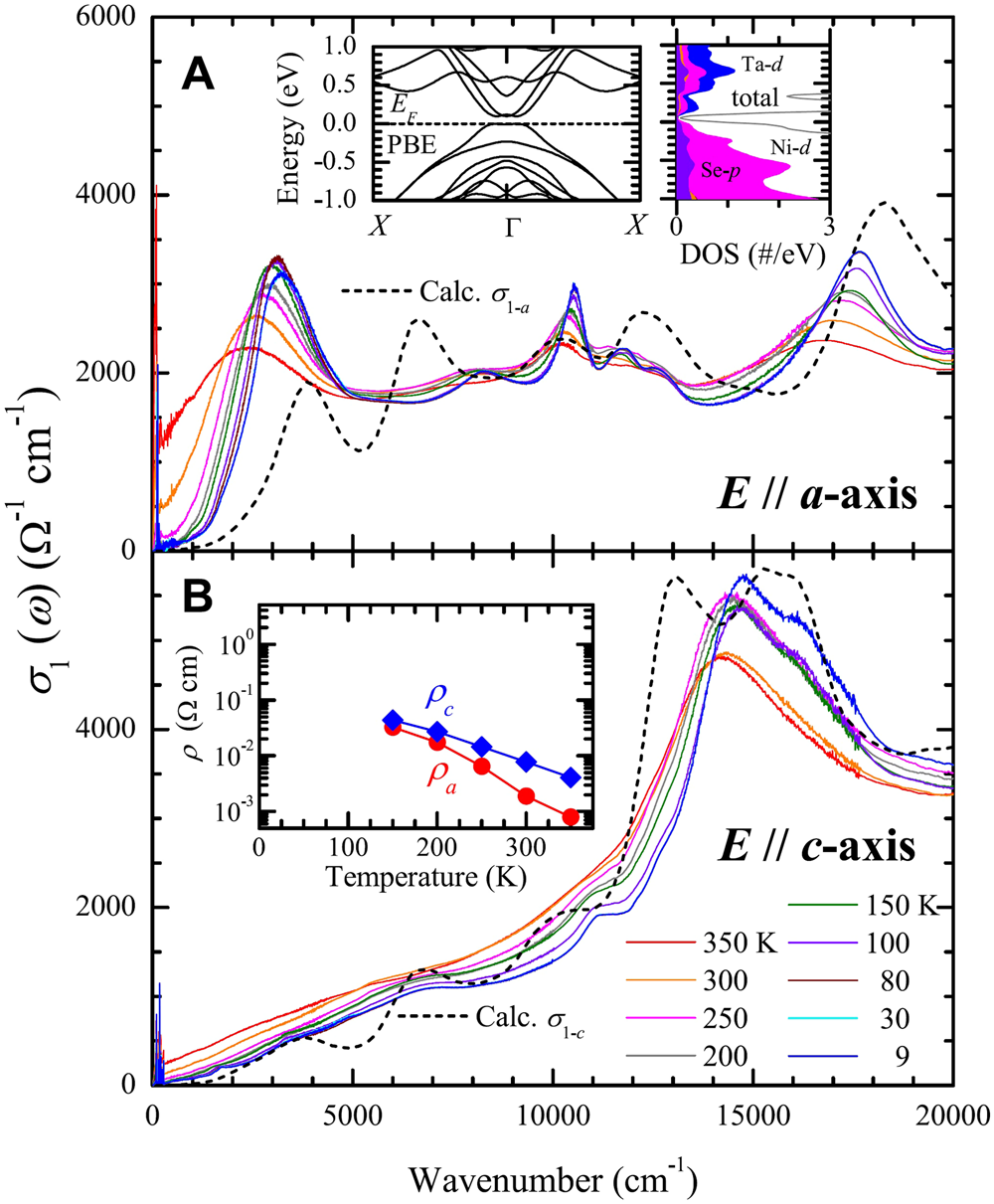
Anisotropic optical conductivity of Ta_2_NiSe_5_ along *a*- and *c*- axes. The experimentally optical conductivities of Ta_2_NiSe_5_ along *a*- and *c*-axes are depicted in (**A**) and (**B**), respectively. The optical conductivities were obtained with the Kramers-Kronig analysis on the measured reflectance spectra for the different temperatures (within the range of 9 to 350 K). The inset of (**A**) shows the band dispersion diagrams obtained via the PBE functional and the partial densities of states, which were obtained from the first-principles calculations. The inset in (**B**), shows the extracted dc resistivity (along *a*- and *c*- axes) from extrapolations of the optical conductivity to zero frequency. The dashed black lines correspond to the theoretical optical conductivities for *a*- and *c*- axis orientations obtained from the first-principles calculations.

**Figure 3:**
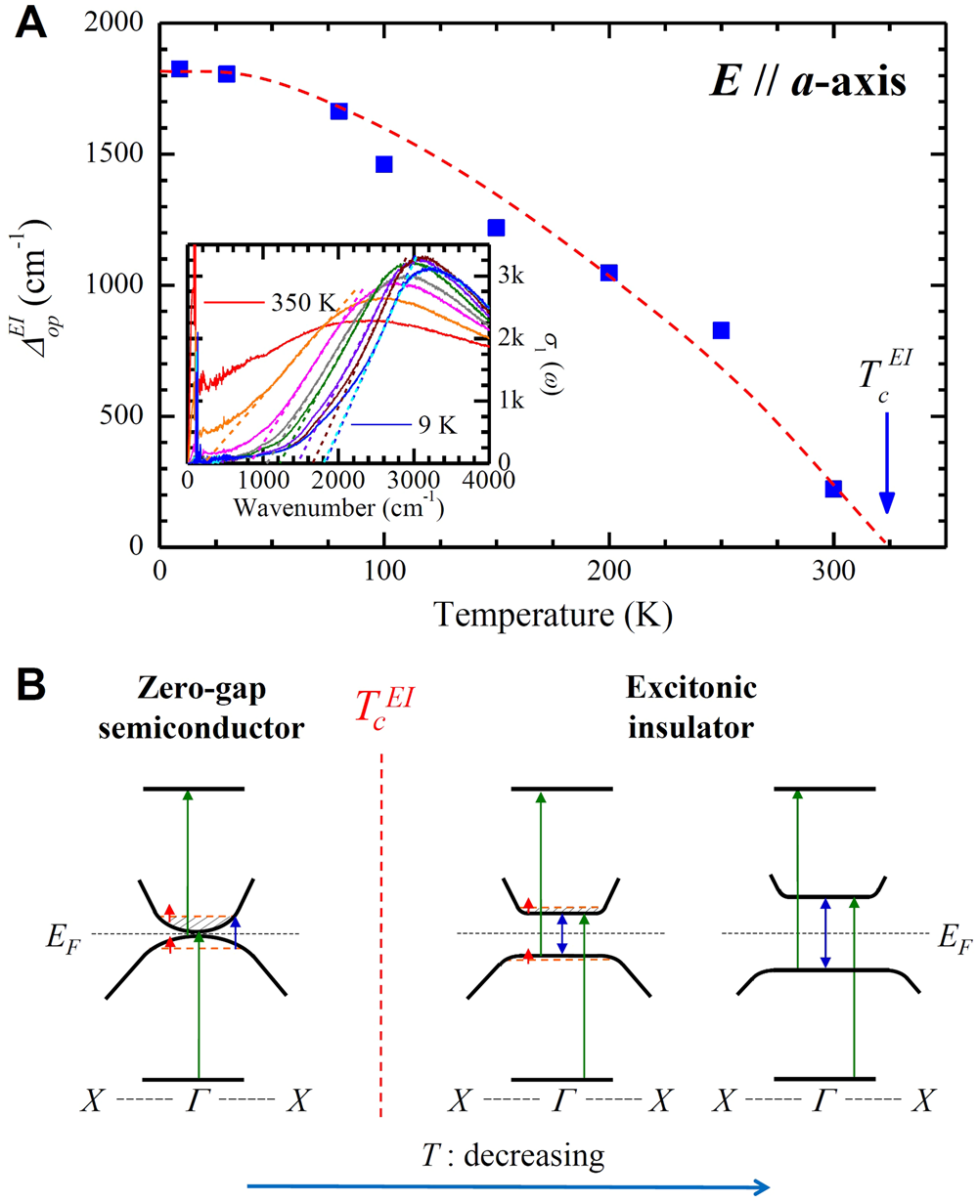
Excitonic insulator energy gap and temperature-dependent interband transitions. (**A**) The temperature-dependent optical EI gap (${{\rm{\Delta }}}_{op}^{EI}$) of Ta_2_NiSe_5_ obtained from the optical conductivity. The red dashed line is a guide to the eye. The inset shows the procedure by which the optical EI gap was obtained. (**B**) A schematic diagram of the temperature-dependent evolution of the optical EI gap in Ta_2_NiSe_5_, above and below the transition temperature (${T}_{c}^{EI}$). The intraband transitions are denoted with red arrows. The upward blue arrow denotes the interband transition between the two parabolic bands near the Fermi level. The double direction blue arrows denote the transitions between the two flat bands, which form the optical EI gap. The other interband transitions involving bands near the Fermi level are depicted with green arrows.

Figure 2(A and B) depict the optical conductivity for the *a*- and *c*-axes of the Ta_2_NiSe_5_ sample, respectively. The optical conductivities were obtained from the measured reflectance using the well-developed Kramers-Kronig analysis^17^. In Fig. 2(A), the optical conductivity of 9 K shows a strong and sharp interband transition (or peak) near 3050 cm^−1^ with an optical gap on the low frequency side of the peak. As the temperature increases, the spectral weight of the peak shifts towards the low frequency region, thereby filling up the optical gap. This temperature-dependent behavior of the peak is similar to a typical signature of an optical gap formation^18^. A detailed discussion and analysis on this optical gap and the temperature-dependence of the 3050 cm^−1^ peak will be covered in the following section. This peak centered near 3050 cm^−1^ seems to be closely related to the flat valence band (or the proposed excitonic condensation feature) near the Γ-point in the Brillouin zone, which was observed via ARPES studies^9,10,13^, since its temperature-dependent behavior and energy scale are similar to those of the flat valence band. The same sharp interband transition has been reported in a recent study of Ta_2_NiSe_5_ probed using spectroscopic ellipsometry^15,16^. Interestingly, we also observe some more sharp peaks in the optical conductivity (associated with interband transitions) in a higher energy region above 5000 cm^−1^, with these peaks displaying a temperature-dependence behavior similar to that of the 3050 cm^−1^ peak. The similar temperature-dependence behavior will also be discussed later (refer to Fig. 3(B) and adjoining discussion). We have also extracted the dc resistivity from the optical conductivity by extrapolation to *ω* = 0. The extracted dc resistivity for both *a*- and *c*- axes of Ta_2_NiSe_5_ single crystal is displayed in the inset of Fig. 2(B). The temperature-dependence profile and relative values of the extracted dc resistivity are consistent with those reported ones in recent literature^15^. The optical conductivity of the Ta_2_NiSe_5_ single crystal along the *c*-axis (depicted in Fig. 2(B)) displays a monotonic temperature-dependence. A strong interband transition peaked near ∼15,000 cm^−1^ was observed, with an energy corresponding to the *d*-*d* transition between the valence Ni-*d* and conduction Ta-*d* orbitals^19^. There was also a significant absorption below this transition, which is not the focus of this article.

First-principles calculations were performed to compute the anisotropic optical properties of Ta_2_NiSe_5_.(refer to the Method) The inset in Fig. 2(A) displays the electronic dispersion along the X-Γ-X direction, which is chosen to compare with the ARPES study results^10,13^. The Fermi energy (*E*_*F*_) is set to the top of the valence band. The Ni-*d* and Ta-*d* orbitals account for the majority of the valence and conduction bands, respectively, as evident from the inset of Fig. 2(A). The results obtained with the mBJ functional were similar to those found with PBE, although the bandgap is twice that obtained with PBE. In general, the results are consistent with those reported by Kaneko *et al*.^11^. We calculated the optical conductivity from the dielectric function obtained using the random phase approximation (RPA)^20^ and our first-principles calculation results. The calculated optical conductivity (*σ*_1−*a*_(*ω*) and *σ*_1−*c*_(*ω*)) for the *a*- and *c*- axes are displayed as dashed black lines in Fig. 2(A and B), respectively. The difference between the two theoretical conductivity spectra is due to the effect of Ta-NiSe-Ta chains along the *a*-axis^7,8^. The calculated *σ*_1−*c*_(*ω*) agrees reasonably with the measured conductivity of the *c*-axis in its overall shape. However, the calculated *σ*_1−*a*_(*ω*) shows some discrepancy in the low frequency region below 5000 cm^−1^; the theoretical conductivity shows a higher energy gap and a much smaller spectral weight as compared to the measured one at 9 K. We speculate that this discrepancy occurs due to the non-inclusion of electron-hole interactions in our calculations. Therefore, this result may indicate that the sharp interband transition near 3050 cm^−1^, is closely related to the excitonic excitations. Our observation is in line with a previous report on carbon nanotubes^21^, wherein a similar set of calculations (with and without including electron-hole interactions) yielded a similar difference between two results. It is important to note that it is not easy to include electron-hole interactions in the electronic structure calculations for a complex system like Ta_2_NiSe_5_.

## Temperature-dependent optical excitonic insulator gap and interband transitions

Figure 3(A) displays the temperature-dependent optical gap in the *a*-axis optical conductivity of single crystal Ta_2_NiSe_5_, which may stem from the formation of excitonic condensation. We call the optical gap as an optical excitonic insulator (EI) gap, which will be the same as the exciton binding energy since the band gap of Ta_2_NiSe_5_ has been known to be zero^15^. The inset depicts the method by which the optical gap is extracted from the optical conductivity data; this is an approximate method in order to see a temperature-dependence of the optical gap. We note that there is some amount of spectral weight below the gap, whose origin is not clearly figured out yet. We observe the optical gap starting to open below ∼325 K which is the onset temperature, ${T}_{c}^{EI}$ marked with an arrow. The extracted gap opening temperature (${T}_{c}^{EI}$) obtained with this approach appears to be almost identical to the structural transition temperature (${T}_{c}^{Str}$). Hence, the two phenomena with the characteristic onset temperatures appear to be closely related to each other. The magnitude of the optical EI gap increases monotonically with decrease in the temperature and we find that ${{\rm{\Delta }}}_{op}^{EI}(T)/{{\rm{\Delta }}}_{0}\simeq 1-{(T/{T}_{c}^{EI})}^{2}$ (refer to the following section). The size of the full gap (Δ_0_) at *T* = 0 is ∼1800 cm^−1^ (or 0.22 eV) and is consistent with previously reported value (∼0.16 eV at 150 K) in recent literature^14^, after accounting for the temperature-dependence. For the Ta_2_NiSe_5_ system, this optical EI gap (or the exciton binding energy) is much larger than exciton binding energies of bulk (or three-dimensional) semiconductors^15^. The extremely large binding energy can be understood if we consider that the excitons in Ta_2_NiSe_5_ exist along the one-dimensional chains, where long-range Coulomb interaction between an electron and a hole can exist^21,22^.

In Fig. 3(B), with the help of schematics, we illustrate the evolution of the electronic structure in the Ta_2_NiSe_5_ sample for temperatures above and below the EI transition temperature (${T}_{c}^{EI}$). Above ${T}_{c}^{EI}$, Ta_2_NiSe_5_ is known as a zero-gap semiconductor (ZGSC)^15^. Therefore, the electronic structure near the Fermi surface can be depicted with two parabolic valence and conduction bands, which are nearly touching each other. Since the temperature is quite large (above $\simeq$325K), we probably have some thermally promoted electrons (or holes) at the bottom of the conduction band (on the top of the valence band), which yields a finite dc conductivity (refer to the red arrows). We expect a broad peak in the optical conductivity at a finite frequency, due to interband transition between the conduction and valence bands near the Fermi level (refer to the blue arrow). We also may have other empty and filled flat bands near the Γ point below and above these parabolic bands as shown in the figure and these bands (refer to the black horizontal lines) may not depend on the temperature. These flat bands probably exist along the quasi-one-dimensional chains and, in fact, the theoretical calculation shows similar flat bands near the Γ point (refer to the inset of Fig. 2(A)). For the ZGSC phase, we expect both intraband and interband transitions from filled states below the Fermi energy to empty states above. These intraband and interband transitions are shown with red arrows, and blue and green arrows, respectively. In fact, we observe these intraband and interband transitions in the measured optical conductivity at 350 K (refer to Fig. 2(A)), which appear as finite dc conductivity and broad peaks, respectively. When the temperature decreases below ${T}_{c}^{EI}$, the optical EI gap (${{\rm{\Delta }}}_{op}^{EI}$) opens and gradually increases, as evident from Fig. 3(A). In this case, the bottom of the valence band and the top of the conduction band are flat and parallel to each other; these flat bands get wider as the temperature is lowered further. Therefore, in the EI phase, a distinct energy gap near the Fermi level, results in a sharp peak just above the gap in the optical conductivity (see in Fig. 2(A)) since the joint density of states may have a singularity. We have other peaks (or interband transitions) in the high frequency region (above 5000 cm^−1^) and observe that these peaks shift to a higher energy and become better defined as the temperature of the sample is lowered. From the schematic, similar temperature-dependence behaviors of all the interband transitions can be rationalized if we consider that the flat bands near the Fermi level induced by the EI phase transition could be involved in the interband transitions in the high frequency region. Our qualitative description of the temperature-dependent behavior of the higher-energy interband transitions is our speculation. It is not completely confirmed by rigorous quantitative analysis yet.

## Temperature-dependent accumulated spectral weight and excitonic superfluid plasma frequency

**Figure 4:**
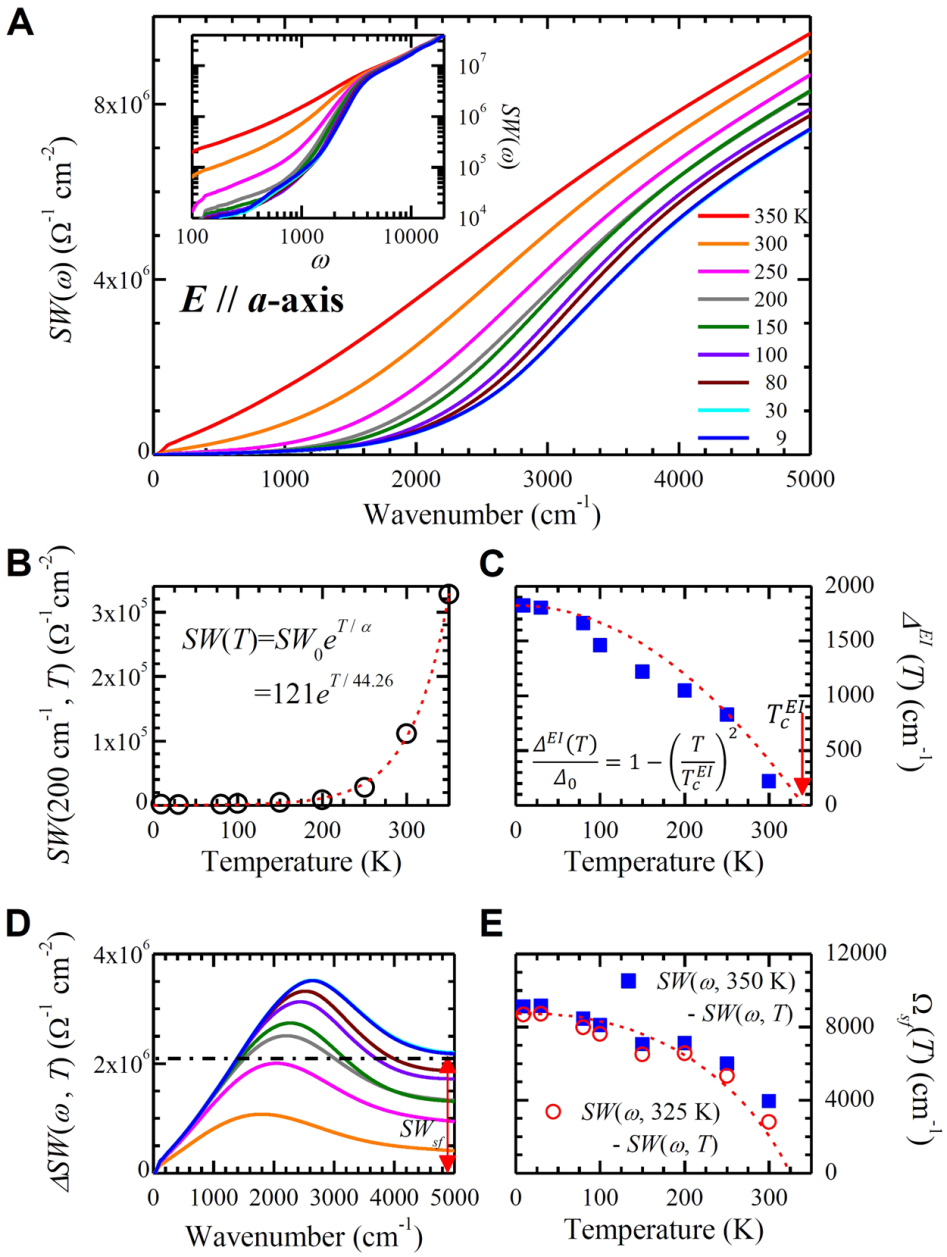
Temperature-dependent accumulated spectral weight and excitonic superfluid spectral weight. (**A**) The accumulated spectral weights of Ta_2_NiSe_5_ along the *a*-axis as a function of frequency at various temperatures. The inset shows the same quantity in log-log scales. (**B**) The accumulated spectral weight at 200 cm^−1^ as a function of temperature. Red dashed line is an exponential fit to the data. (**C**) The EI energy gap (${{\rm{\Delta }}}^{EI}(T)$) as a function of temperature obtained considering the thermal effects (see the text for a detailed description) and the optical EI gap (${{\rm{\Delta }}}_{op}^{EI}(T)$) extracted from the optical conductivity (refer to Fig. 3(A)). The equation (${{\rm{\Delta }}}^{EI}(T)/{{\rm{\Delta }}}_{0}=1-{(T/{T}_{c}^{EI})}^{2}$) is for the red dashed line with ${T}_{c}^{EI}=338$ K. The red arrow indicates ${T}_{c}^{EI}$. (**D**) A quantity, ${\rm{\Delta }}SW(\omega ,T)\equiv SW(\omega ,\,350K)-SW(\omega ,T)$. The red arrow shows the missing spectral weight which is closely related to the superfluid spectral weight. (**E**) Extracted excitonic superfluid plasma frequency (Ω_*sf*_ (*T*)) as a function of temperature shown with solid blue squares. The open red circles are adjusted excitonic superfluid plasma frequency (see in the text). The dashed line is a guide to the eye for the open circles.

In general, an optical gap formation results in spectral weight redistributions in the optical conductivity. We studied the spectral weight redistribution of the first interband transition peaked near 3050 cm^−1^ with respect to both frequency and temperature. The accumulated spectral weight (*SW*) is a useful physical quantity for studying the spectral weight redistribution and can be defined as $SW(\omega ,T)\equiv {\int }_{{0}^{+}}^{\omega }{\sigma }_{1}(\omega ^{\prime} ,T)d\omega ^{\prime}$. In Fig. 4(A), *SW*(*ω*) of the Ta_2_NiSe_5_ sample are displayed at various temperatures in a frequency range up to 5,000 cm^−1^. All the accumulated spectral weights were observed to be more or less parallel to one another above ∼4000 cm^−1^ while a small amount of suppression in the accumulated spectral weight occurred below ∼4000 cm^−1^, for temperatures below the transition temperature (${T}_{c}^{EI}$). In the inset, *SW*(*ω*) for a wider spectral range up to 20,000 cm^−1^ in log-log scales is displayed.

In Fig. 4(B), *SW*(*ω*) at 200 cm^−1^ as a function of temperature is shown. We chose a low frequency of 200 cm^−1^, well below the full optical EI gap (1800 cm^−1^) to study only the thermal excitation effect, excluding other absorptions at high frequencies. It is important to note that we have subtracted 10,000 Ω^−1^ cm^−2^ from the obtained *SW*(200*cm*^−1^, *T*) to exclude contributions from the low frequency phonons. We show an exponential fit (red dashed line) to the data; the equation employed in the fit is $SW(T)\simeq S{W}_{0}\exp (T/\alpha )=121\exp (T\mathrm{/44.26}K)$, where *SW*_0_ is the spectral weight up to 200 cm^−1^ at *T* = 0 K. Since this accumulated spectral weight at low frequency is proportional to the charge carrier concentration due to a predominant thermal effect, it can be written as $SW(T)\simeq S{W}_{0}\exp \{\,-\,[{{\rm{\Delta }}}^{EI}(T)-{{\rm{\Delta }}}_{0}]/T\}$, where ${{\rm{\Delta }}}^{EI}(T)$ is an excitonic insulator (EI) energy gap and Δ_0_ ≡ Δ^*EI*^(0). By combining these two equations, we obtain ${{\rm{\Delta }}}^{EI}(T)/{{\rm{\Delta }}}_{0}=1-{T}^{2}/({{\rm{\Delta }}}_{0}\alpha )=1-{(T/{T}_{c}^{EI})}^{2}$ ($\ge 0$), where (${T}_{c}^{EI}=\sqrt{{{\rm{\Delta }}}_{0}\alpha }$) is the onset temperature of the gap. If we consider Δ_0_ as the full optical EI gap (≃1800 cm^−1^) and *α* = 44.26 (from the exponential fit), then the onset temperature (${T}_{c}^{EI}$) is 338 K. In Fig. 4(C), we plot the EI energy gap, Δ^*EI*^(*T*), with the red dashed line along with the optical EI gap, ${{\rm{\Delta }}}_{op}^{EI}(T)$, which was obtained directly from the optical conductivity (refer to Fig. 3(A)). From the figure, it is evident that these two results are in good agreement with each other.

Furthermore, from the accumulated spectral weight, we calculate an interesting physical quantity, the superfluid plasma frequency of the excitonic condensation. In Fig. 4(D), we present a differential quantity, ${\rm{\Delta }}SW(\omega ,T)\equiv SW(\omega ,\,350K)-SW(\omega ,T)$ at various temperatures. This quantity seems to consist of two components: one is unrecovered spectral weight near 5000 cm^−1^ (or the missing spectral weight) due to the excitonic condensation marked with the red arrow and the other is the peak near 2600 cm^−1^ due to the thermal broadening effects, which may come from two different temperatures of the two different phases (here, the most pronounced peak between 9 K and 350 K). We present a more detailed discussion on this quantity (Δ*SW*(*ω*, *T*)), comparing it with that of the superconductors, in the following section. The superfluid spectral weight (*SW*_*sf*_) can be related to the superfluid plasma frequency (Ω_*sf*_) as ${{\rm{\Omega }}}_{sf}(T)\equiv \sqrt{\mathrm{(120/}\pi )\,S{W}_{sf}(T)}$. We note that the numerical factor *π*/120 is the unit conversion factor; here Ω_*sf*_ and *SW*_*sf*_ are in cm^−1^ and Ω^−1^ cm^−2^ units, respectively. We display the superfluid plasma frequency (blue solid squares) as a function of temperature in Fig. 4(E). Ω_*sf*_ is gradually decreasing with increasing the temperature and then eventually going to zero near the EI onset temperature, ${T}_{c}^{EI}$. Therefore, the onset temperature of the superfluid condensation seems to be the same as that of the EI energy gap. It is worthwhile to note that up to now we used the spectral weight at 350 K as the reference spectral weight for getting the differential spectral weight at various temperatures since we do not have data closer to the transition temperature. If we use a linear interpolated spectral weight (at 325 K) between 300 K and 350 K as the reference spectral weight we will have slightly lower excitonic condensation plasma frequencies than the values obtained using the spectral weight at 350 K as the reference spectral weight, as displayed in Fig. 4(E) with red open circles.

## Discussion: Excitonic insulators and superconductors

**Figure 5:**
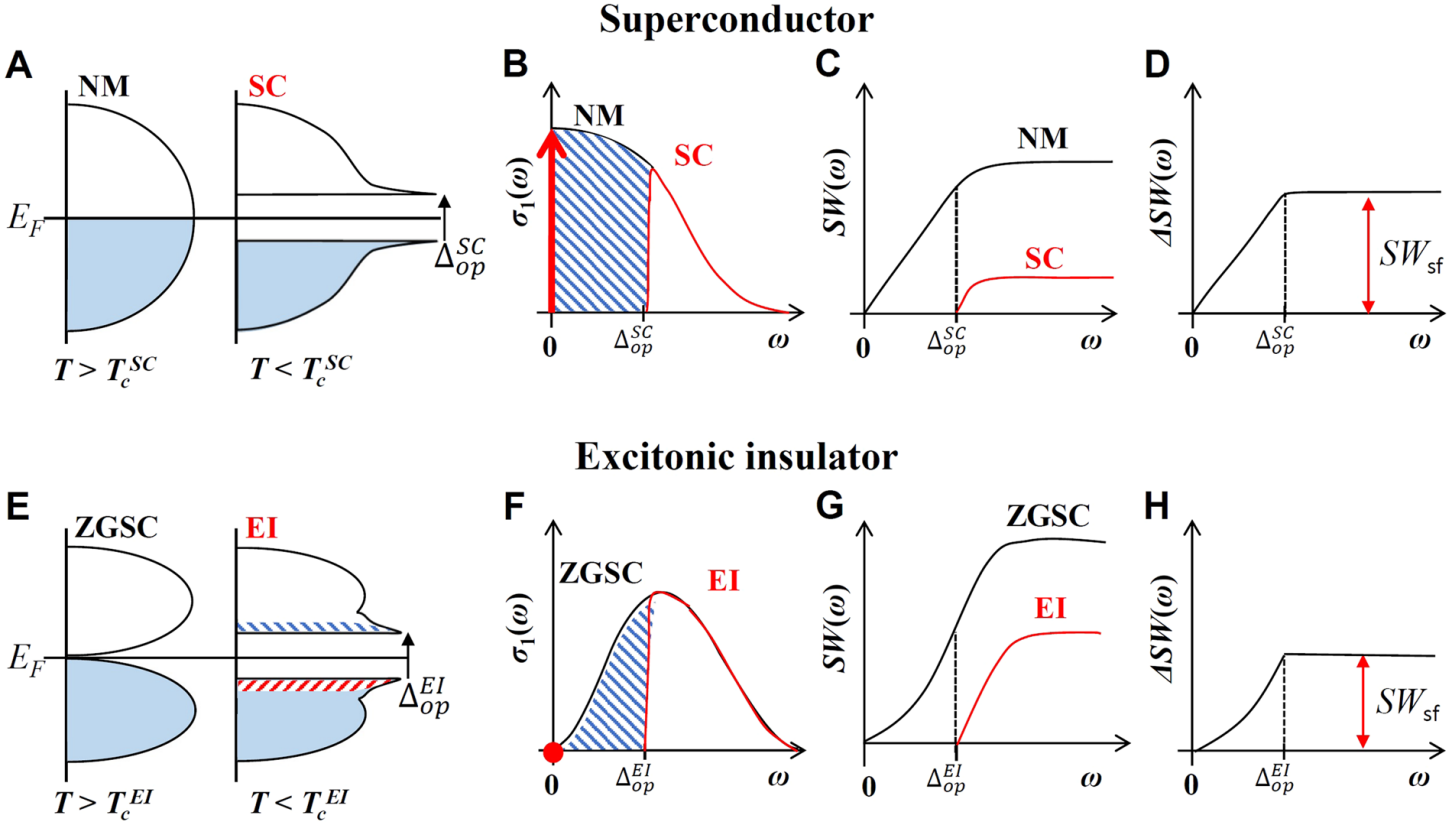
Comparison of excitonic insulators with superconductors. (**A**) The densities of states (DOS’s) of normal metal (NM) and superconductor (SC). (**B**) The corresponding optical conductivities (*σ*_1_(*ω*)). The thick red vertical arrow indicates the superfluid spectral weight condensed at zero frequency. (**C**) The accumulated spectral weights (*SW*(*ω*)) of NM and SC. (**D**) The differential spectral weight (Δ*SW*(*ω*)) between NM and SC. (**E**) DOS’s of zero-gap semiconductor (ZGSC) and excitonic insulator (EI); the red (electrons) and blue (holes) dashed horizontal lines show the thermal excitations, (**F**) *σ*_1_(*ω*); the black (for ZGSC) and red (for EI), (**G**) *SW*(*ω*), and (**H**) Δ*SW*(*ω*). The red arrow shows the missing (or superfluid) spectral weight, which is closely related to the excitonic superfluid plasma frequency.

It is worthwhile to compare the condensation in the EI with that in a superconductor. In Fig. 5(A–H), we compare the two material systems: *s*-wave superconductors (SC) and EI schematically. We depict the transition from a normal metal (NM) to a SC and a zero-gap semiconductor (ZGSC) to an EI, as the temperature is lowered from above to below the transition temperatures (${T}_{c}^{SC}$ and ${T}_{c}^{EI}$), respectively, through spectral weight redistributions. Figure 5(A) shows the density of states (DOS’s) of the normal metal and the superconductor, 5(B) shows the corresponding optical conductivities (*σ*_1_(*ω*)), 5(C) shows the accumulated spectral weights (*SW*(*ω*)), and 5(D) shows the differential spectral weights (${\rm{\Delta }}SW(\omega )\equiv SW(\omega ,T > {T}_{c}^{SC})-SW(\omega ,T < {T}_{c}^{SC})$). In the superconductor, the superfluid spectral weight of condensed Cooper (or electron-electron) pairs appears as a delta function at zero frequency, which is marked with a thick red vertical arrow in Fig. 5(B); the hatched area below the superconducting gap (${{\rm{\Delta }}}_{op}^{SC}$) seems to have disappeared^23^. Therefore, this area is termed as the missing spectral weight. At high frequencies well above the SC gap, the accumulated spectral weights of NM and SC will differ by the missing spectral weight, as shown in Fig. 5(C). When the condensed Cooper pairs are broken by thermal or other processes, the missing spectral weight reappears back in a finite frequency region. The differential spectral weight (Δ*SW*(*ω*)) in the high frequency region will clearly show the missing spectral weight, as depicted in Fig. 5(D), where *SW*_*sf*_ stands for the superfluid spectral weight.

We also sketch the corresponding four physical quantities (DOS’s, *σ*_1_(*ω*), *SW*(*ω*), and Δ*SW*(*ω*)) for both the ZGSC and the EI in Fig. 5(E–H). In the EI, there is a superfluid spectral weight associated with the condensed electron-hole pairs; however, the neutral excitonic superfluid will be located at zero frequency with zero spectral weight since the neural excitons cannot contribute to the electrical conductivity^4^. The electrons involved in the condensation will be disappeared in the EI states as in the SC state; this also causes a missing spectral weight. However, the electrons in the EI state do not appear anywhere in the whole frequency range while the electrons in the SC state will appear at zero frequency as a delta function. Therefore, in the EI state the optical sum rule seems to be violated; the missing spectral weight (or missing electron density) still remains in the sample but is just optically invisible. The EI has singularities at the bottom of the conduction band and on the top of the valence band, similar to the superconductor, as evident in Fig. 5(E). These singularities are a signature of the condensation, which leads to the flat valence band in ARPES dispersion and a sharp peak in the optical conductivity as shown in Fig. 2(A) and sketched in Fig. 5(F). The singularity at the bottom of the conduction band consists of the electron states while the singularity on the top of the valence band consists of the hole states (illustrated with two different colors in Fig. 5(E)). Here we assume that both ZGSC and EI phases are at the absolute zero temperature. The resulting accumulated spectral weight in Fig. 5(G) looks analogous to what was observed in our measured accumulated spectral weights in Fig. 4(A), for frequencies below 5000 cm^−1^. However, they appear to be different in the high frequency region well above the EI gap. The accumulated spectral weights of ZGSC and EI flatten and are parallel to each other at the high frequency region while the measured ones are still parallel to each other but keep increasing. This difference at the high frequency region can be explained by considering other interband absorptions occurring at higher frequencies. If we include the other interband absorption bands at higher frequencies, the results will show a continuous parallel increase at high frequencies, as in the measured data (refer to Fig. 4(A)). In Fig. 5(H) we display the differential spectral weight (Δ*SW*(*ω*)), where *SW*_*sf*_ stands for the superfluid spectral weight. We do not see the peak, which was observed in the experimental differential spectral weight (refer to Fig. 4(D)). If we take the thermal broadening effects, which may come from the temperature difference between ZGSC and EI phases, into account we will have the peak in the Δ*SW*(*ω*).

## Conclusion

In conclusion, we observed a strong and sharp peak around 3050 cm^−1^ in the optical conductivity (along the *a*-axis) of Ta_2_NiSe_5_ at low temperatures along with a well-defined optical gap on the low frequency side of the peak. This peak corresponds to an interband transition, which shows a characteristic strong temperature-dependence, a behavior previously attributed to that of the flat valence band in observations made of Ta_2_NiSe_5_ with ARPES^9,10,13^. The results of our first-principles calculations were in good agreement with the overall experimental optical data for both *a*- and *c*-axes except for the strong and sharp peak in the *a*-axis conductivity. We speculate that this discrepancy between experiment and theory arises from the fact that the electron-hole interactions in Ta_2_NiSe_5_ were not included in the theoretical calculations. This result probably indicates that the strong and sharp peak results from the electron-hole interactions for this system. Furthermore, the spectral weight redistribution analysis demonstrates that the excitonic condensation of electron-hole pairs can occur below the onset temperature of the optical gap and the temperature-dependent excitonic superfluid plasma frequency can be obtained from the measured optical data. We also found that the optical sum rule can be violated in the EI phase. These interesting findings illustrate the new opportunities for further investigations on Ta_2_NiSe_5_, and other excitonic insulators.

## Experiments and analysis

A high-quality single crystal Ta_2_NiSe_5_ sample was grown by a chemical vapor transport method. The detailed crystal growth method can be found in a literature^14^. The crystal structure and chemical compositions of the Ta_2_NiSe_5_ sample were characterized using x-ray diffraction and energy-dispersive x-ray spectroscopy. Our optical study was performed on the sample with an area of 2 × 2 mm^2^ and a thickness of 0.5 mm. A commercial FTIR-type spectrometer (Vertex 80 v, Bruker) and a continuous flow liquid helium cryostat were used to obtain *a*- and *c*-axis reflectance spectra over a wide spectral range (80–20,000 cm^−1^) at various selected temperatures between 9 and 350 K. We used linear polarized beam to get anisotropic optical spectra with an incident angle on the sample of 10°. We also used an *in-situ* metallization method to obtain accurate reflectance spectra^24^. In this method we used the coated 200 nm thick gold for mid- and far-infrared (or aluminum for near-infrared and visible) film on the sample as the reference reflectance. Furthermore, we corrected the measured reflectance with respect to the gold (or aluminum) film by multiplying the absolute reflectance of the gold (or aluminum). The optical conductivity is obtained from the measured reflectance (*R*(*ω*)) using the Kramers-Kronig relation between the amplitude ($\sqrt{R(\omega )}$) and phase (*ϕ*(*ω*)) of the reflection coefficient^17^, the Fresnel formula, and well-known relationships between the optical constants^25^.

## First-principles calculation

We adopted the full-potential linearized augmented plane wave (FP-LAPW) implemented in Wien2k^26^ to calculate the band structure with a number of exchange-correlation functionals, including the generalized gradient approximation (GGA), GGA + U, van der Waals force correction (vdW)^27^, and their hybrid functionals. We obtained insulating ground-states when we used two GGAs: Perdew, Burke, and Ernzerhof (PBE)^28^ and modified Becke-Johnson (mBJ)^29^. We found that the electronic ground state was semi-metallic when the experimental lattice constants were used^7^; therefore, we fully relaxed the crystal structure using the PBE and then used the relaxed geometry in further calculations for the band structure and optical conductivity. Note that the volume of our relaxed structure is nearly 14% larger than the experimental volume. This difference due to the distance between layers (*b* lattice constant) as shown in the inset of Fig. 1(A): (i) the relaxed *b* lattice constant is 14.459 Å, which is nearly 13% larger than the experimental one. (ii) the a and c lattice constants are 3.509 Å (+0.4%) and 15.732 Å (+0.6%), respectively. The relaxed structure was chosen because our calculated electronic properties are similar to previous *ab-initio* properties found using the experimentally determined lattice constants^11^. The reciprocal space integration was approximated by sampling the Brillouin zone with a 28 × 6 × 28 mesh of the Monkhorst-Pack scheme.
