# Publisher Correction: Temperature-dependent excitonic superfluid plasma frequency evolution in an excitonic insulator, Ta_2_NiSe_5_

**DOI:** 10.1038/s41598-020-61010-5

**Published:** 2020-03-03

**Authors:** Yu-Seong Seo, Man Jin Eom, Jun Sung Kim, Chang-Jong Kang, Byung Il Min, Jungseek Hwang

**Affiliations:** 1 Department of Physics, Sungkyunkwan University, Suwon, Gyeonggi-do 16419 Republic of Korea; 2 Department of Physics, Pohang University of Science and Technology, Pohang, 37673 Republic of Korea

Correction to: *Scientific Reports* 10.1038/s41598-018-30430-9, published online 10 August 2018

The original version of this Article contained errors.

In the title of the paper, the word “superfluid” was incorrectly given as “superuid”. Additionally, the author Byung Il Min was incorrectly indexed.

Furthermore, within the Abstract

“With regard to the *a*-axis optical conductivity, a sharp peak near 3050 cm^−1^ at 9 K, with a well-defined optical gap ${(}_{op}^{EI}\simeq 1800\,{{\rm{cm}}}^{-1})$ and a strong temperature-dependence, is observed. With an increase in temperature, this peak broadens and the optical energy gap closes around ~325 K $({T}_{c}^{EI})$. The spectral weight redistribution with respect to the frequency and temperature indicates that the normalized optical energy gap (${(}_{op}^{EI}(T){/}_{op}^{EI}({\rm{o}}))$) is $1-{(T/{T}_{c}^{EI})}^{2}$.”

now reads:

“With regard to the *a*-axis optical conductivity, a sharp peak near 3050 cm^−1^ at 9 K, with a well-defined optical gap (${\varDelta }_{op}^{EI}\simeq 1800\,{{\rm{cm}}}^{-1}$) and a strong temperature-dependence, is observed. With an increase in temperature, this peak broadens and the optical energy gap closes around ~325 K (${T}_{c}^{EI}$). The spectral weight redistribution with respect to the frequency and temperature indicates that the normalized optical energy gap (${\varDelta }_{op}^{EI}$(*T*)/${\varDelta }_{op}^{EI}$(0)) is $1-{(T/{T}_{c}^{EI})}^{2}$.”

Finally, in the Introduction under subheading ‘Temperature-dependent accumulated spectral weight and excitonic superfluid plasma frequency’,

“If we consider Δ_0_ as the full optical EI gap (≃1800 cm^−1^) and *α* = 44.26 (from the exponential fit), then the onset temperature (*T*_*c*_^*EI*^) is 338 K.”

now reads:

“If we consider Δ_0_ as the full optical EI gap (≃1800 cm^−1^) and *α* = 44.26 K (from the exponential fit), then the onset temperature (*T*_*c*_^*EI*^) is 338 K.”

These errors have now been corrected in the PDF and HTML versions of the Article.

